# Downregulated expression of HSP27 in human low-grade glioma tissues discovered by a quantitative proteomic analysis

**DOI:** 10.1186/1477-5956-8-17

**Published:** 2010-03-26

**Authors:** Guobo Shen, Shufang Liang, Zhizhong Xu, Liangxue Zhou, Shiyin Xiao, Xiangying Xia, Ronghui Li, Ying Liao, Chao You, Yuquan Wei

**Affiliations:** 1 State Key Laboratory of Biotherapy and Cancer Center, West China Hospital, West China Medical School, Sichuan University, Chengdu 610041, China; 2 Department of Neurosurgery, West China Hospital, West China Medical School, Sichuan University, Chengdu 610041, China; 3 Department of Biochemistry and Molecular Biology, West China Medical School of Preclinical and Forensic Medicine, Sichuan University, Chengdu 610041, China

## Abstract

**Background:**

Heat shock proteins (HSPs), including mainly HSP110, HSP90, HSP70, HSP60 and small HSP families, are evolutionary conserved proteins involved in various cellular processes. Abnormal expression of HSPs has been detected in several tumor types, which indicates that specific HSPs have different prognostic significance for different tumors. In the current studies, the expression profiling of HSPs in human low-grade glioma tissues (HGTs) were investigated using a sensitive, accurate SILAC (stable isotope labeling with amino acids in cell culture)-based quantitative proteomic strategy.

**Results:**

The five HSP family members were detected and quantified in both HGTs and autologous para-cancerous brain tissues (PBTs) by the SILAC-based mass spectrometry (MS) simultaneously. HSP90 AB1, HSP A5(70 KDa), and especially HSP27 were significantly downregulated in HGTs, whereas the expression level of HSPA9 (70 KDa) was little higher in HGTs than that in PBTs. It was noted that the downregulation ratio of HSP27 was 0.48-fold in HGTs *versus *PBTs, which was further validated by results from RT-PCR, western blotting and immunohistochemistry. Furthermore, we detected HSP27 expression changes along with cell growth under heat shock treatment in glioma H4 cells.

**Conclusion:**

The SILAC-MS technique is an applicable and efficient novel method, with a high-throughput manner, to quantitatively compare the relative expression level of HSPs in brain tumors. Different HSP family members have specific protein expression levels in human low-grade glioma discovered by SILAC-MS analysis. HSP27 expression was obviously downregulated in HGTs *versus *PBTs, and it exhibited temporal and spatial variation under heat shock treatment (43°C/0-3 h) *in vitro*. HSP27's rapid upregulation was probably correlated with the temporary resistance to heat shock in order to maintain the survival of human glioma cells.

## Background

Heat shock proteins (HSPs) are evolutionary conserved proteins involved in various cellular processes. According to their different molecular weights, structure and functions, HSPs contain major five groups, including the HSP110/HSP100, HSP90, HSP70, HSP60 and small HSP families [1]. The expressions of HSPs are induced under the existence from oxidative stresses, heavy metals, osmotic stresses, metabolic poisons, viral/microbial infections and other external stresses [1,2]. HSPs have multiple intracellular functions, including protein chaperoning [3], signal transduction, modulation of immune response [4], differentiation and proliferation [5], as well as regulation of apoptosis [6]. In tumor cells, HSPs are also involved in the resistance to therapeutic agents [7,8].

However, the amino acids and biological functions differ among individual HSP family and different members [3]. For example, heat shock protein 27 (HSP27), also called HSP B1, belongs to the small HSP family with a 27-KDa molecular weight, which is found to express from prokaryotes to mammals [9]. HSP27 has potential to interact with several proteins [1,9], including as a multiple inhibitor of apoptosis [10-12]. HSP27 can negatively regulate cell death by interacting with cytochrome c [13], and it can also activate protein kinase B and Akt, which inhibit cell death by phosphorylating and inactivating procaspase-9 [14].

Previous studies have indicated that HSP27 expression is implicated in prognosis in many cancers, however it has a different prognostic indication for different types of tumors. The prognosis is poor when HSP27 expresses in ovarian [15], gastric [16], liver [17] and prostate cancer [18], as well as osteosarcoma [19]. Oppositely, HSP27 expression suggests a good prognosis in endometrial adenocarcinoma, oesophageal cancer and malignant fibrous histiocytoma [20]. By now, most of studies on HSP expression in brain tumor tissues have been obtained from conventional methods, such as immunohistochemistry [21-23] or two-demensional gel eletrophoresis [24]. Currently, novel proteomic techniques, especially the stable isotope labeling with amino acids in cell culture (SILAC)-based quantitative proteomics [25], offer possibility of systematically investigating the expression profiling of different HSP members in glioma in terms of quantitative view. Because the SILAC-based quantitative proteomics is a powerful tool in distinguishing the isoform-specific expression profiling in tissues, by which *in vivo *tagging of an isotope-labeling amino acid in proteins assists quantitative comparisons for differentially expressed proteins in different pathological tissues [26-28].

In the current report, by using this novel proteomic approach, we investigated the differentital expression profiling of HSPs between human glioma tissues (HGTs) and autologous para-cancerous brain tissues (PBTs). Besides, we further focused on the expression variations of HSP27 by heat shock in a glioma-derived cell line H4, as well as analyzed the relationship between HSP27 expression and cell growth. These findings presented the first quantitative proteomic analysis on HSPs in HGTs. The lower expression of HSP27 in glioma tissues is probably associated with cell differentiation in human glioma development, and the rapidly upregulation of HSP27 against heat shock *in vitro *may have a temporary potential to increase survival ability of glioma cells.

## Results

### Labeling course for incorporation of Leu-d3 in H4 cells

**Figure 1:**
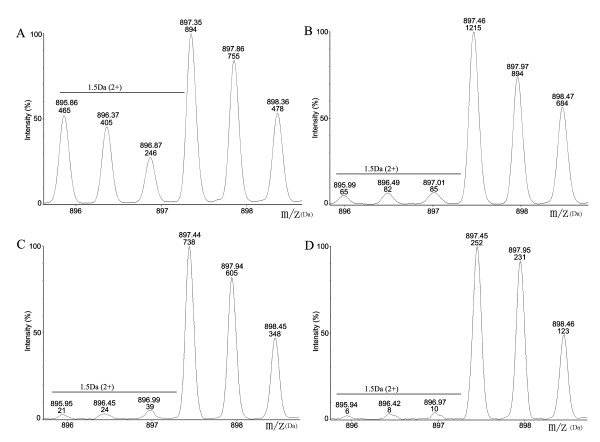
**Incorporation of Leu-d_3 _in β-actin at different passages of H4 cells**. A-D represented samples respectively from 1, 3, 5 and 7 passages of labeled H4 cells. The represented pairs of peptides "**SYE*L*PDGQVITIGNER" **from β-actin were analyzed at different time points by ESI-TOF/MS. The intensity ratio of pairs of isotope peaks (m/z, 897/895) was respectively about 65.78%, 94.92%, 97.23% and 97.67%. After seven passages, the incorporation of Leu-d_3 _was exceed 97.5%, which indicated that the labeling was complete.

A time course was performed to detect the labeling efficiency which was required for cells to incorporate the deuterated-leucine (Leu-d_3_) fully into all proteins. As described in our previous papers [26,29,30], β-actin was usually chosen to detect the labeling efficiency. For example, we detected the incorporation ratio of Leu-d_3 _labeling in a representative Leu-containing peptide (SYE*L*PDGQVITIGNER) of β-actin. Because the normal L-leucine (Leu-d_0_) was gradually replaced with the Leu-d_3 _into β-actin during cell growth, the ratio of peptide intensity of the Leu-d_3_-labeling peptide *versus *Leu-d_0_- containing peptide was steadily increased (Figure 1). A larger incorporation of Leu-d_3 _was observed at later time points with full incorporation after the 5th cell passage, and the incorporation rate of Leu-d_3 _exceeded 97%. Thus the labeling H4 cells were harvested at least after five cell passages to extract proteins for the following SILAC and mass spectrometric analysis.

### Protein expression profiling of HSPs in human glioma tissues

Because the original SILAC approach is directly applied in cell system, we have expanded it for tissue proteome quantification indirectly based on Leu-d_3_-labeling proteins from a cultured glioma-derived cell line H4 as internal standards [27]. Corresponding to the low-grade glioma tissues in our present study, H4 is a neuroglioma cell line derived from a low-grade malignant glioma [31-33] to take as an internal standard for protein relative quantification in MS [27], which was proved to express all of HSP proteins those were identified in human glioma tissues. The principle for protein quantification and comparison between two states of tissues by SILAC-MS had been portrayed in our previous papers [26-28].

In order to normalize the internal standard for comparison, we first compared the expression level of housekeeping protein β-actin in HGTs and PBTs. As shown in the Table 1, the SILAC ratio was close to 1.00 either in HGTs or in PBTs, which suggested that it expressed with similar levels between these two states of brain tissues. This was also indicated that the total protein mixture from labeling H4 cells and HGTs was equal to that quantity mixed from labeling H4 cells and PBTs, and there was no need for additional normalization to compare.

**Table 1:** The expression quantification of β-actin and HSPs by the unique Leu-containing isotope peptides in MS

		MOWSE scores(b) (Queries Match)		**SILAC-Ratio(e) (mean ± SD; No. of Pep**^**f**^**)**		Unique Leu-containing peptide coverage(%)		
Protein name	IPI no.(a)	HGTs (c)	PBTs (d)	Ratio1	Ratio2	HGTs (c)	PBTs (d)	Change-Ratio(g) (ratio1/ratio2)
**HSP110/100 family**								
HSP H1(105 KD)	IPI00218993	77(8)	140(10)	0.61 ± 0.02 (2)	0.82 ± 0 (1)	2.7	1.6	0.74
								
**HSP90 family**								
HSP90 AB1	IPI00334775	428(18)	179(10)	0.29 ± 0.17 (6)	0.45 ± 0.26 (3)	8.7	4.8	0.64
HSP90 AA1	IPI00382470	82(14)	109(11)	0.80 ± 0.43 (4)	0.75 ± 0.52 (2)	6	3	1.07
								
**HSP70 family**								
HSP A8(71 KD)	IPI00003865	486(16)	360(17)	0.52 ± 0.09 (8)	0.55 ± 0.1(3)	17	5	0.95
HSP A9(70 KD)	IPI00007765	401(16)	127(5)	0.49 ± 0.09 (5)	0.34 ± 0 (1)	9.6	2.2	1.44
HSP A5(70 KD)	IPI00003362	508 (15)	99(8)	0.33 ± 0.15 (7)	0.57 ± 0.28 (2)	14	3.2	0.58
								
**HSP60 family**								
HSP D1(60 KD)	IPI00472102	67(9)	419(20)	0.57 ± 0.08 (3)	0.52 ± 0.27(4)	33	18	1.10
								
**Small HSP family**								
HSP B1(27 KD)	IPI00025512	159(12)	138 (10)	0.29 ± 0.12 (4)	0.61 ± 0.32 (3)	8.2	7	0.48
								
**Control protein**								
β-actin	IPI00021439	687(31)	643(39)	1.03 ± 0.02 (4)	0.98 ± 0.03 (4)	12	12	1.05

(a) IPI (International Protein Index) no. is the protein accession number, which was derived from the EMBL-EBI database.

(b) 'MOWSE' is derived from molecular weight search, and it is a method for the identification of proteins from the molecular masses of peptides created by proteolytic digestion and measured by MS.

(c) The protein mixture from HGTs and Leu-d_3_labeling H4 cells; HGTs: human glioma tissues.

(d) The protein mixture from PBTs and Leu-d_3_labeling H4 cells; PBTs: para-cancerous brain tissues.

(e) SILAC ratio1 (HGTs/H4) and SILAC ratio2 (PBTs/H4): the peak intensity ratio of Leu-d_0_-labeling peptides from HGTs or PBTs *versus *that of Leu-d_3_-labeling peptides from H4 cells, which was quantified using Analyst QS software.

(f) The number of unique Leu-containing peptides of one protein to calculate SILAC ratio1 and SILAC ratio2.

(g) The Change-Ratio between two tissues (SILAC Ratio1/SILAC Ratio2).

**Figure 2:**
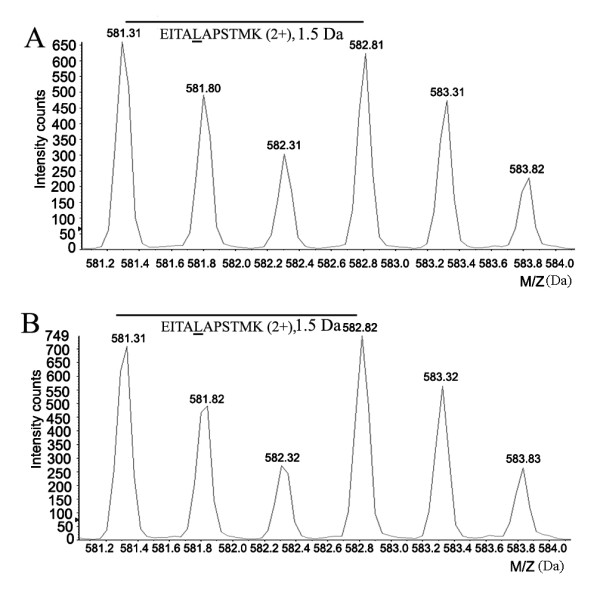
**One pair of isotope peptides (m/z, 582/581) "EITA*L*APSTMK" of β-actin was chosen to be an internal standard to validate the mixture ratio**. (A) showed the peak intensity of the isotope-labeling peptides from the protein mixture containing human glioma tissues (HGTs) and H4 cells. The SILAC ratio1 of HGTs *versus *labeled H4 cells was 1.03 ± 0.02. (B) represented the peak intensity of isotope-labeling peptides, which came from the protein mixture from para-cancerous brain tissues (PBTs) and labeled H4 cells. The SILAC ratio2 of PBTs *versus *labeled H4 cells was 0.98 ± 0.03. Thus, the change ratio of β-actin in HGTs *versus *PBTs was 1.05, near 1:1, which indicated the expression of β-actin was similar between the two tissues.

**Figure 3:**
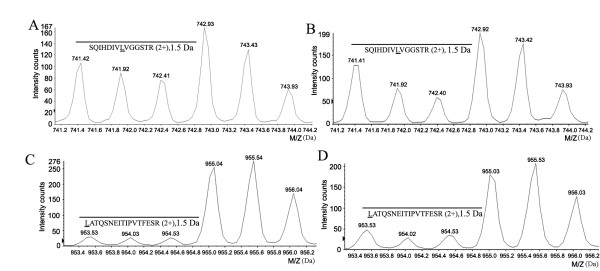
**The representative pairs of isotope labeling peptides to quantify HSP expression in glioma tissues**. (A)-(B) was the peak intensity of isotope labeling peptide "**SQIHDIV*L*VGGSTR" **of HSPA8(71 KD) respectively from HGTs/H4 (A) and PBTs/H4(B) samples. A: the SILAC ratio1 (HGTs/H4) of HSPA8 was 0.52 ± 0.9; B: the SILAC ratio2 (PBTs/H4) of HSPA8 was 0.55 ± 0.1. The change ratio (HGTs/PBTs) was 0.95 (0.52/0.55) indicated HSPA8 was no significant change between glioma tissues and para-cancerous brain tissues. Similarly, the pairs of isotope labeling peptide "***L*ATQSNEITIPVTFESR**" of HSP27(HSPB1) from HGTs/H4 (C) and PBTs/H4(D) samples for quantitation, and the change ratio of 0.48 (0.29/0.61) showed HSP27 was down-regulated in glioma tissues.

The relative expression levels of HSPs were summarized as Table 1 based on the change-ratio of SILAC Ratio1 *versus *SILAC Ratio 2 (Ratio1/Ratio2), which was obtained from the average of two biological replicate experiments. The average relative standard deviation (RSD) of the SILAC ratio was 0.16 for all quantified HSP proteins. Based on our quantitative criteria established [26-30], in our present study, the cut-off selection of change ratio is set 2-fold RSD (0.32) as the threshold. In other words, we assigned the ratio of up-regulation over 1.32 and the down-regulation below 0.68 for both altered proteins. Therefore, the HSP27 (IPI00025512), HSP A5 (IPI00003362) and HSP90AB1 (IPI00334775) respectively had 0.48, 0.58 and 0.64-fold downregulation in HGTs compared with that in PBTs. And HSPA9 (IPI00007765) was increased with 1.44-fold in HGTs, while the other HSP members, listed in the Table 1, exhibited no significant differences between HGTs and PBTs. The representative pairs of isotope labeling peptides for quantification were illustrated as Figure 2 and 3, respectively from the control β-actin, a down-regulated protein HSP27 and an unchanged HSPA8.

### Expression validation of HSP27 in human glioma tissues

**Figure 4:**
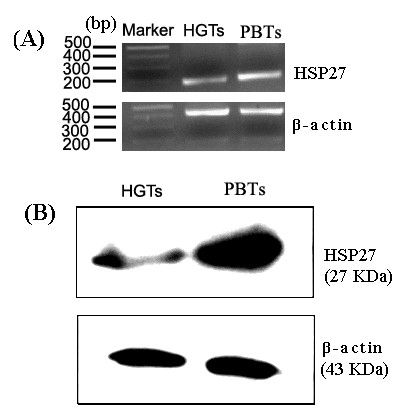
**The expression validation of HSP27 in glioma tissues by RT-PCR (A) and Western blot (B)**. HGTs: human glioma tissues; PBTs: para-cancerous brain tissues. Marker: DNA Marker; The β-actin was taken as a loading control.

In order to validate the expression level of HSP27 by the quantitative proteomic analysis, the expression status of HSP27 in mRNA and protein levels were compared by a semi-quantitative RT-PCR and western blotting between glioma and para-cancerous brain tissues. As shown in the Figure 4, compared with the expression level in PBTs, HSP27 was decreased in glioma tissues both at mRNA (Figure 4A) and protein levels (Figure 4B). Three cases of HGTs and PBTs were performed repeatedly, and same results were obtained.

**Figure 5:**
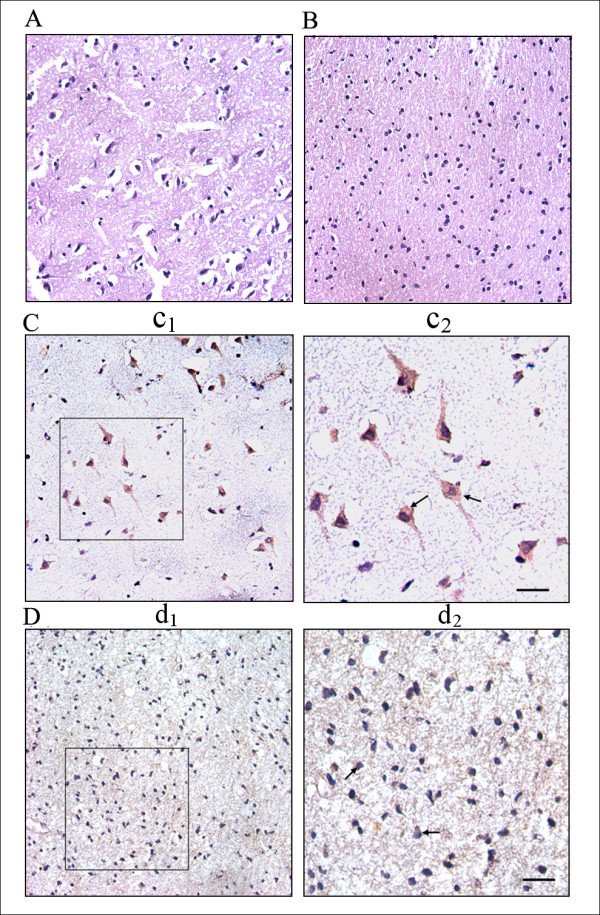
**The expression and distribution of HSP27 in human glioma and para-cancerous brain tissues**. (A) the HE staining of para-cancerous brain tissues, which have typical characterization of normal brain tissues; (B) HE staining of glioma tissues. HSP27 immunoreactivity in the representative para-cancerous brain tissue (C) and glioma tissue (D) (original magnification ×400). The positive staining of HSP27 in the neuronal somata and proximal processes was shown as yellow-brown granules indicated by arrows. The figure c_2_-d_2 _was the insert of its corresponding expanded figure c_1_-d_1_. The scale bar was 10 μm.

We further analyzed the expression and distribution of HSP27 between two types of brain tissues by immunohistochemistry (IHC). As shown in Figure 5, the para-cancerous brain tissues had intensive HSP27 immunoreactivity in neuroplasm of the neuronal somata and proximal processes (Figure 5-C2). While under same conditions, the relative expression level of HSP27 was much lower in glioma tissues (Figure 5-D2). All these data were consistent with the above quantitative proteomic results.

### The correlation between the expression variation of HSP27 and cell growth under heat shock treatment

**Figure 6:**
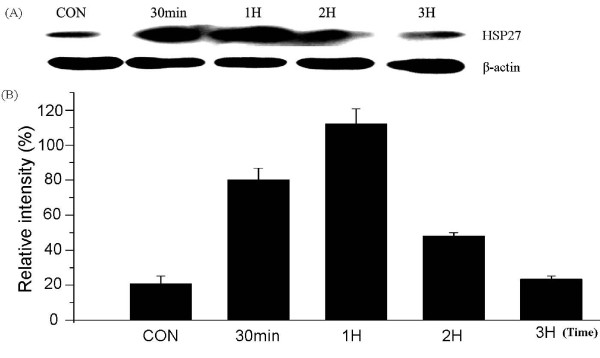
**The expression variation of HSP27 protein under heat shock (A)**. The relative strength of band was determined based on the control by Quality-One software(Bio-Rad) (B). Compared with the control, HSP27 expression was increased to 3.83 and 5.36-fold at time point of 30 min and 1 h after heat shock, then gradually jumped back near to the background level at 2 h and 3 h, with 2.3 and 1.12-fold expression *versus *the control. CON: the control cells without heat shock.

The previous reports indicated that HSP27 overexpression *in vivo *exhibited neuroprotective properties by HSP27-mediated inhibition of apoptosis in neuronal cells [34]. Based on these reports and our current data, we speculated whether the lower expression of HSP27 in glioma could regulate cell survival *via *its anti-apoptotic effect. Therefore, we further analyzed the correlations of its expression with cell growth under heat shock in H4 cells. As presented in Figure 6, the expression of HSP27 exhibited temporal and spatial variations under heat shock treatment (43°C)for 0-3 h. Compared with the untreated control cells, HSP27 expression was increased about 3.83 and 5.36-fold at 30 min and 1 h after heat shock, then gradually jumped back near to the background level at the time point of 2 h and 3 h, respectively with 2.3 and 1.12-fold expression level *versus *the control.

**Figure 7:**
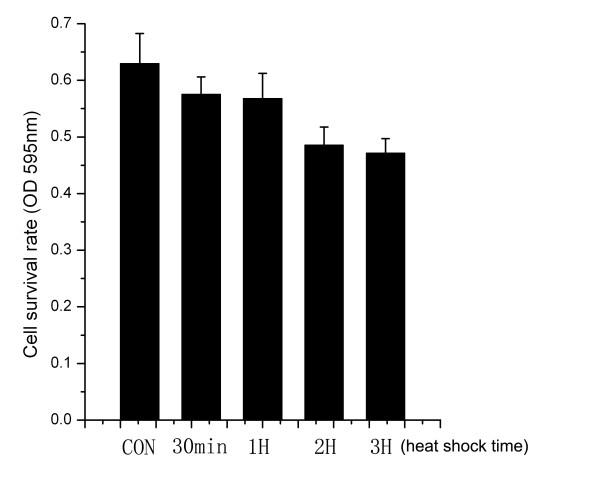
**The survival rate of H4 cells with heat shock**. It was noticed that cell proliferation ability under heat shock at the time point of 30 min(OD_595 nm_, 0.57 ± 0.03, n = 18) was almost similar with that at 1 h (OD_595 nm_, 0.57 ± 0.04, n = 18), and a same status was observed at the time point of 2 h(OD_595 nm_, 0.48 ± 0.03, n = 18) and 3 h (OD_595 nm_, 0.47 ± 0.03, n = 18) treatment. However, at the time point of 0.5-1 h and 2-3 h treatment, compared with control (OD_595 nm_, 0.63 ± 0.05, n = 18), cell growth had significantly decreased (p < 0.01) in these two groups. CON: the control cells without heat shock.

In order to investigate whether there were some relationships between HSP27 expression and cell growth *in vitro *under heat shock, cell growth was detected by 3-(4,5-dimethylthiazol- z-y1)-2,5-diphenyltetrazolium bromide (MTT). It was noticed that cell proliferation ability under heat shock at the time point of 30 min(OD_595 nm_, 0.57 ± 0.03, n = 18) was almost similar with that at 1 h (OD_595 nm_, 0.57 ± 0.04, n = 18), which indicated that a rapid increase of HSP27 expression in H4 cells can exhibit resistance to a transitory external stress (hyperthermia). The cell survival number was changed little at the time point of 2 h (OD_595 nm_, 0.48 ± 0.03, n = 18) and 3 h (OD_595 nm_, 0.47 ± 0.03, n = 18) treatment. However, at each treatment period during 0.5-1 h and 2-3 h, compared with control group (OD_595 nm_, 0.63 ± 0.05, n = 18), cell growth had significantly decreased (p < 0.01) in these two groups. Therefore, under heat shock treatment within 0-3 h, cell survival rate was gradiently diminished corresponding to the variation of HSP27 expression in H4 cells (Figure 7).

## Discussion

The hotspot chaperone molecules -- heat shock proteins, have been disclosed to be tightly associated with the brain tumors, and the expression variations of HSPs imply the progressive staging and prognosis of glioma [20-24,35], which provides a novel attempt to develop the utility of a specific HSP as the diagnotic target and therapeutic intervention [24,35]. Therefore, in current study, we mainly applied the SILAC-based proteomics to compare the expression differences of HSPs between human low-grade glioma tissues and the autologous PBTs. HSP27 was identified to have lower expression levels in HGTs, which was validated not only by RT-PCR, western blot and IHC data, but also by previous other's reports [21-23], including in Hermission's paper [23].

More importantly, this SILAC-based quantitative proteomic method provides several new information of HSP family proteins in HGTs by investigating protein expression profiling. Firstly, the SILAC-MS technique can precisely take 'in-spectra'quantitative measurement of expressing profiles of HSPs in different states with a high-throughput manner according to the ratio of isotopic peaks derived from labeling proteins, which principle has been delineated in detail and applied to distinguish members belonging to a homologous protein family in our recent studies [26-28]. The five main members of HSP family were successfully identified and quantified in HGTs simultaneously by the SILAC-aided LC/MS-MS analysis, and their expression levels were different in glioma. Especially, HSPA9 (70 KD) was 1.44-fold upregulation in HGTs, whereas the HSP90AB1, HSPA5 and HSP27 all had lower expression level in HGTs than that in PBTs. Although HSPA9 and HSPA5 both belong to the same HSP70 family, HSPA9 is increased in HGTs, whereas HSPA5 is decreased. The SILAC-MS method offers a sensitive and quick view of expression characterization for HSP family than other traditional protein-by-protein assessment, such as by the relative strength of IHC/Western blot [21-23].

Secondly, we put emphasis on comparison with the relative expression levels of HSPs between low-grade HGTs (grade II) and their counterparts -- autologous PBTs by an accuratly quantitative proteomic method, especially revealed the different expression changes of HSP27 in the pathologic state, which provides valuble clues in diagnosis and therapeutic intervention for giloma. While Hermission et al. had just detected the expression of HSPs in 12 human glioma cell lines by Western blot and in glioma tissues by a qualitative IHC [23]. It has been found that the expression of HSP27 in human astrocytomas correlates with histologic grades and tumor growth fractions. In the low-grade astrocytomas, the HSP27 immunopositive rate was very low, but the positive rate was significantly higher in the high-grade astrocytomas [21]. Our quantitative data on HSP27 expression in low-grade glioma also supported the previous results, which were obtained by a semiquantitative immunohistochemistry fashion.

Besides, we further investigated that the correlations between the expression variation of HSP27 and cell growth *in vitro *under heat shock treatment. Transient hyperthermia (43°C/0.5-2 hours) induced a rapidly increased expression of HSP27 in H4 cells, especially it accessed to the maximal with 5.36-fold expression by heat treatment at 43°C for 1 h, whereas the cell growth was almost not affected at the time point of 30 min and 1 h by heat shock. This indicated that the increased expression of HSP27 by heat shock treatment in H4 cells can exhibit resistance to a transitory external stress, hyperthermia-induced cell damage, to keep cell growth and survival. However, in our current studies, the mechanism against heat shock is not explored in detail so far. For example, how did the expression variation of HSP27 regulate cell survival signaling, and it cooperated with other heat shock proteins, including HSPA9, HSP90AB1 and HSPA5? And what were the expression levels of the other three HSPs under the same heat shock treatment? Future studies on these issues will help us to understand the functional significance of these HSPs in glioma.

## Materials and methods

### Leu-d_3 _labeling

Human glioma cell line H4 was cultured in isotope-labeling 1640 media with 10% dialyzed fetal bovine serum (FBS, GIBCO), which was supplemented with the Leu-d_3 _(L-leucine-5,5,5-D3, 98%; Cambridge Isotope Laboratories, Inc, U.K.) replaced normal Leu-d_0_. The labeling H4 cells were cultured at 37°C with 5% CO_2_, and grown at least for five passages until complete labeling for protein identification and quantification. The culture media were changed every 3 days, and cell pellets were respectively collected for every cell passage to monitor the labeling ratio like as our previous reports [26,29,30].

### Tissues

HGTs and PBTs were obtained from West China Hospital, Sichuan University of China with the offers' informed consent. Each sample was identified through pathologic biopsy. PBTs, which show typical characterization of normal brain tissues, are taken as the normal brain tissues. The glioma tissues used in our studies were identified as the astrocytoma with a grade II. All tissues were gained immediately after surgical operation, and stored in the liquid nitrogen. The study was approved by the local institutional ethics committee.

### Antibodies

HSP27 monoclonal antibody was purchased from Cell Signaling Technology, Inc. (G31, #2402, Cell Signaling Technology, Inc., USA). The β-actin antibody (sc-1616, Santa Cruz, USA) was taken as a control for western blot. The secondary antibodies were purchased from Abcam (Abcam inc., USA).

### Protein extraction

Different passages of H4 cells were collected, cell pellets and tissue samples were respectively treated to extract total proteins by our previous method [26]. The protein concentration was quantified using the Protein Assay Kit (#500-0006, Bio-Rad, USA).

### Protein separation and in-gel digestion

In order to detect the labeling efficiency of H4 cells, proteins were separated by SDS-PAGE and stained with Coomassie blue (R-250, Bio-Rad, USA). The band of β-actin was excised, subjected to in-gel tryptic digestion. The peptides were resolved in 50% acetonitrile/0.1% trifluoroacetic acid and analyzed by the electrospray ionization-time of flight (ESI-TOF) mass spectrometry with reverse liquid chromatography (LC) (Waters Corporation, USA).

To identify and quantify HSP expression profiles in two different states of brain tissues by the SILAC-MS technique, 20 μg proteins from Leu-d_3_-labeling H4 cells were respectively mixed with same quantity of proteins from HGTs and PBTs, then the protein mixture was separated on 12% SDS-PAGE and stained by Coomassie blue (R-250). Gels were excised and subjected to in-gel digestion by MS-grade trypsin (#V5113, Promega, USA), peptides were identified by LC-MS/MS mainly as described as following.

### Protein identification and quantification by LC-MS/MS

Protein identification was performed by LC-nanospray-MS/MS analysis using a QSTAR XL mass spectrometer (Applied Biosystems, USA). The MASCOT server was used to interpret the LC-MS/MS data by searching against human proteins from EMBL-EBI database (IPI-human-335). The parameters of database search had been described before [26,27].

The expression level of a specific heat shock protein was quantified by tracking its unique pairs of Leu-containing peptides from mass spectra. The SILAC ratio of one protein was defined as the relative intensity ratio of Leu-d_0_-containing peptides from each kind of tissues *versus *that of Leu-d_3_-labeling peptides from H4 cells, and it was averaged when several peptides were used for quantification for one protein. The SILAC ratio1 was represented the ratio of isotope peaks from the protein mixture of HGTs with labeled H4 cells (HGTs/H4 cells), and the SILAC ratio2 was the ratio of isotope peaks from the protein mixture of PBTs with labeled H4 cells (PBTs/H4 cells). Therefore, the relative expression levels (change ratio) of HSPs in HGTs *versus *PBTs were estimated by calculating the ratio of their SILAC ratios (SILAC ratio1/SILAC ratio2).

### Semiquantitative RT-PCR

Total RNA was isolated using Trizol reagent (Invitrogen) to take as template to amplify each target cDNA fragment. The primers of HSP27 and β-actin for RT-PCR were designed as following. The forward primer for HSP27 was 5'-TCC CTG GAT GTC AAC CAC TTC G-3' (22 bp), reverse primer was 5'-GGG ACA GGG AGG AGG AAA CTT G-3' (22 bp), and the length of the amplified cDNA was 184 bp. The designed primers for β-actin was 5'-CGG GAA ATC GTG CGT GAC-3'(18 bp, forward), 5'-TGG AAG GTG GAC AGC GAG G-3' (19 bp, reverse), and the length of the amplified cDNA was 434 bp.

The PCR reaction was performed in 25 μl system, which contained 2.5 μl PCR- buffer, 1.5 μl MgCl_2_, 2 μl dNTPs, 1 μl of 10 pmol forward and reverse primer, 2 μl cDNA template synthesized by using the RevertAid™ First Strand cDNA Synthesis Kit (#K1622, Fermentas, USA), 0.5 μl rTaq polymerase (Takara), and 11.5 μl H_2_O. The reaction was performed as follows: first cycle at 95°C for 2 min, and then 30 cycles at 94°C for 45 s, 54°C for 1 min, 72°C for 1 min and a final extension cycle of 72°C for 5 min. 5 μl of PCR products were identified by 1.2% agar electrophoresis, stained with Goldview (Sigma, USA), and visualized by UV fluorescence. The house-keeping gene β-actin was taken as a loading control.

### Western blotting and immunohistochemistry

Each of 30 μg protein from the tissues (HGTs and PBTs) or treated cells was separated on 12% SDS-PAGE and transferred to PVDF membrane to incubate with HSP27 antibodies to detect the protein expression level, and the β-actin was taken as the loading control.

The paraffin-embedded PBTs and HGTs were cut into slices with 4 μm thickness for Hematoxylin-Eosin (HE) and IHC analysis. The autologous para-cancerous brain tissues were used for the positive control of HSP27 expression. The primary HSP27 antibodies, 1:500 diluted in PBS, was applied for IHC.

### Heat shock treatment on H4 cells

Human glioma H4 cells were cultured in media 1640 with 10% FBS. After cells grew to 70% confluence, the media were carefully removed. Cells were washed gently with sterile PBS and then overlaid with pre-warmed (43°C) media. Cells were incubated in an incubator with 43°C for 0 min, 30 min, 1 h, 2 h and 3 h respectively. After cells were treated by heat shock, the old media were replaced with the new, and cells were incubated in 37°C for another 24 h to collect for western blotting. The H4 cells without heat shock were used as the control.

### Effects of heat shock on cell growth

H4 cells were seeded into 96-well plates at a density of 3-4 × 10^3^/well. When cell confluence was about 70%, cells were washed gently with sterile PBS and then overlaid with pre-warmed (43°C) media. Cells were treated by heat shock at 43°C respectively for 0 min, 30 min, 1 h, 2 h and 3 h described as above, then were incubated for 24 h at 37°C, and 20 μl of MTT (5 mg/ml in PBS) (Sigma) was added to incubate for another 4 h. The medium was removed, and 200 μl DMSO was added to dissolve MTT precipitate. After agitating cells on orbital shaker for 10 min, the absorbance was detected on a spectra max MS (MDC, USA) at 595 nm with a reference filter at 630 nm. The H4 cells without heat shock were used as the control.

### Data statistical analysis

All quantitative data were calculated as mean ± SD from at least two repeated biological experiments. Comparisons between two groups were performed by Student's t test, comparisons among multiple groups were performed by One-way ANOVA and Dunnet-t test. The statistical significance was defined as P < 0.05. Computations were determined by the SPSS 11.5 software package.

## Abbreviations

ESI-TOF: electrospray ionization-time of flight; FBS, fetal bovine serum; HE: Hematoxylin-Eosin;HGTs, human glioma tissues; IHC: immunohistochemistry; LC: liquid chromatography; Leu-d_0_: L-leucine;Leu-d_3_, deuterated-leucine (L-leucine-5,5,5-D3); MS: mass spectrometry; MTT: 3-(4,5 -dimethylthiazol- z-y1)-2,5-diphenyltetrazolium bromide; PBTs: para-cancerous brain tissues; SILAC: stable isotope labeling with amino acids in cell culture; HSPs: heat shock proteins; HSP27: heat shock protein B1(27 KDa).

## Competing interests

The authors declare that they have no competing interests.

## Authors' contributions

SG performed the experiments and wrote the paper draft; LS conceived, designed the experiments and revised the paper; XZ and ZL performed some experiments and analyzed partial data; XS and YC collected and validated tissue samples; XX, LR and LY prepared cell samples and cooperated some experiments with SG; WY supervised the project and provided experimental devices.
